# Inter-Rater Agreement in the Assessment of Video Recordings of Eye Drop Instillation by Glaucoma Patients

**DOI:** 10.1371/journal.pone.0145764

**Published:** 2016-01-05

**Authors:** Meghan S. Park, Marguerite M. Patel, Daniel Sarezky, Carin Rojas, Clara Choo, Michael Choi, Dachao Liu, Alfred W. Rademaker, Angelo P. Tanna

**Affiliations:** 1 Department of Ophthalmology, Northwestern University Feinberg School of Medicine, Chicago, IL, United States of America; 2 Scheie Eye Institute, University of Pennsylvania, Philadelphia, PA, United States of America; 3 Department of Ophthalmology, University of Arizona, Tucson, AZ, United States of America; 4 Department of Ophthalmology, Mayo Clinic, Rochester, MN, United States of America; 5 Department of Preventive Medicine, Northwestern University Feinberg School of Medicine, Chicago, IL, United States of America; University of East Piedmont, ITALY

## Abstract

**Purpose:**

To create a standardized method for evaluating the video recordings of patients self-instilling eye drops and to determine the level of agreement of eye drop instillation efficacy, safety and efficiency ratings by three masked graders.

**Design:**

Prospective cross-sectional study.

**Participants:**

78 patients with open-angle glaucoma or ocular hypertension who had at least 6 months of experience with the use of eye drop medications.

**Methods:**

Participants were video recorded while self-instilling artificial tears sequentially to both eyes. Three masked observers graded these video recordings on three criteria: efficacy (the determination of whether an eye drop was instilled on the ocular surface), safety (assessment of whether the tip of the medication bottle made contact with the ocular surface or eyelids), and efficiency (the number of eye drops expressed from the bottle).

**Main Outcome Measures:**

After grading the video recordings based on efficacy, safety, and efficiency, kappa statistics were used to estimate inter-rater agreement.

**Results:**

The mean kappa level of agreement for efficacy, safety, and efficiency was 0.64 (95% confidence interval (CI), 0.42–0.87), 0.73 (95% CI, 0.58–0.88), and 0.62 (95% CI, 0.42–0.81), respectively.

**Conclusions:**

We demonstrated good inter-rater reproducibility of the masked analysis of video recordings of patients self-instilling eye drops based on three criteria: efficiency, safety, and efficacy.

## Introduction

Current treatment for glaucoma relies heavily on the self-administration of ocular hypotensive eye drops. Several studies have examined the various reasons underlying improper instillation of eye drops by patients, including poor visual acuity [1], older age [2–4], and physical comorbidities [2]. One of the main barriers to glaucoma eye drop compliance is the physical coordination required to, under ideal circumstances, express a single drop such that it lands on the ocular surface without the tip of the bottle making contact with the eye, eyelids or face. The ability to properly administer an eye drop requires visualizing [5] and squeezing the bottle [1, 3], and also detecting whether the drop has landed on the eye. Studies that have video recorded patients administering their eye drops have found that a large proportion of patients do not correctly instill their eye drops in that they miss the conjunctival sac [6] or use multiple drops when only one is needed [2–3, 5].

Furthermore, patients may contaminate the bottle tip or sustain trauma through touching the ocular surface or eyelids. Many glaucoma patients may have other co-morbid ocular conditions that may predispose them to eye infections such as the presence of a trabeculectomy bleb, for example. Geyer, et al [7] reported that the bottle tip was more contaminated than the eye drops themselves, allowing transfer of microorganisms if the bottle tip touches the patient’s ocular surface.

In using more than one drop, patients may run out of eye drops earlier, and thus may need a medication refill before the insurance company will cover it. If a patient cannot financially cover the cost of paying for a medication out-of-pocket [8], the patient may use the medication less frequently or run out and stop using a medication completely until the next refill is covered. This would lead to further problems of nonadherence due to improper dosing. Furthermore, there may be more long-term side effects such as increased lid pigmentation from a prostaglandin analog if an excessive number of drops are used or if the medication is inadvertently applied to the eyelid [9–10].

We propose a three-part classification system for the evaluation of eye drop self-instillation, in which determinations are made as to whether an eye drop landed on the ocular surface (efficacy), whether there was contact between the tip of the bottle and the ocular surface or eyelids (safety), and how many drops were expressed (efficiency). Although previous studies have variably assessed similar outcomes using video recordings, those studies did not attempt to validate the methodology. The goal of this study is to validate the use of video recordings to measure efficacy, safety and efficiency of eye drop self-instillation by assessing inter-rater agreement by three masked graders.

## Materials and Methods

This study was approved by the Northwestern University Institutional Review Board. Written informed consent was obtained from each participant. The study followed the tenets of the Declaration of Helsinki, and is in compliance with the Health Insurance Portability and Accountability Act of 1996.

Patients aged 40–85 years old with open-angle glaucoma or ocular hypertension who had at least 6 months of experience with ocular hypotensive eye drops were sequentially recruited for the study at the time of their clinical office visits. To be included in the study, patients had to self-administer 1–3 eye drop medications daily at home, and have a best corrected visual acuity of 20/50 or better in each eye. Exclusion criteria included moderate to severe cognitive defects as defined by a mini mental status exam score < 20, or the presence of a clinically significant tremor. Once participants fulfilled the inclusion-exclusion criteria and gave informed consent, they were video recorded while self-instilling artificial tears. The video camera was a digital HD recorder, with 42X zoom. Participants were given an opened, sterile bottle of artificial tears (10 mL), and instructed to instill 1 drop onto each eye, in their typical manner, while being recorded. All participants were given access to a mirror and/or a reclining chair to mimic their usual practice of eye drop instillation if they desired. No practice sessions were allowed and the first attempt was recorded. The research assistant was allowed to film the patient in any view and zoom she deemed allowed her the best view of the eye bottle tip and ocular surface.

Three masked graders (MSP, MP, DS) independently scored the 156 video recordings of 78 participants based on efficacy, safety, and efficiency using a forced choice protocol. The videos were viewed on a 13 inch Macbook Pro at 0.5X speed. Graders were medical students who were familiar with the literature on glaucoma medication compliance and were trained using sample video recordings. Graders were allowed to replay the videos an unlimited number of times. Efficacy was graded in a binary fashion: the participant either successfully instilled at least one drop on the ocular surface or failed to do so. Safety was also graded in a binary fashion: the bottle tip either did or did not make contact with the ocular surface or periocular skin. Efficiency was graded as the number of drops expelled from the bottle (from 0 to a maximum of 5). Participants who instilled a continuous stream of eye drops were given the maximum score of 5.

Kappa statistics were used to estimate inter-rater agreement. Weighted kappa was used for inter-rater agreement of efficiency. Analyses were done separately for right and left eyes. Kappa levels and their confidence intervals were then averaged across the three raters and two eyes.

## Results

**Fig 1:**
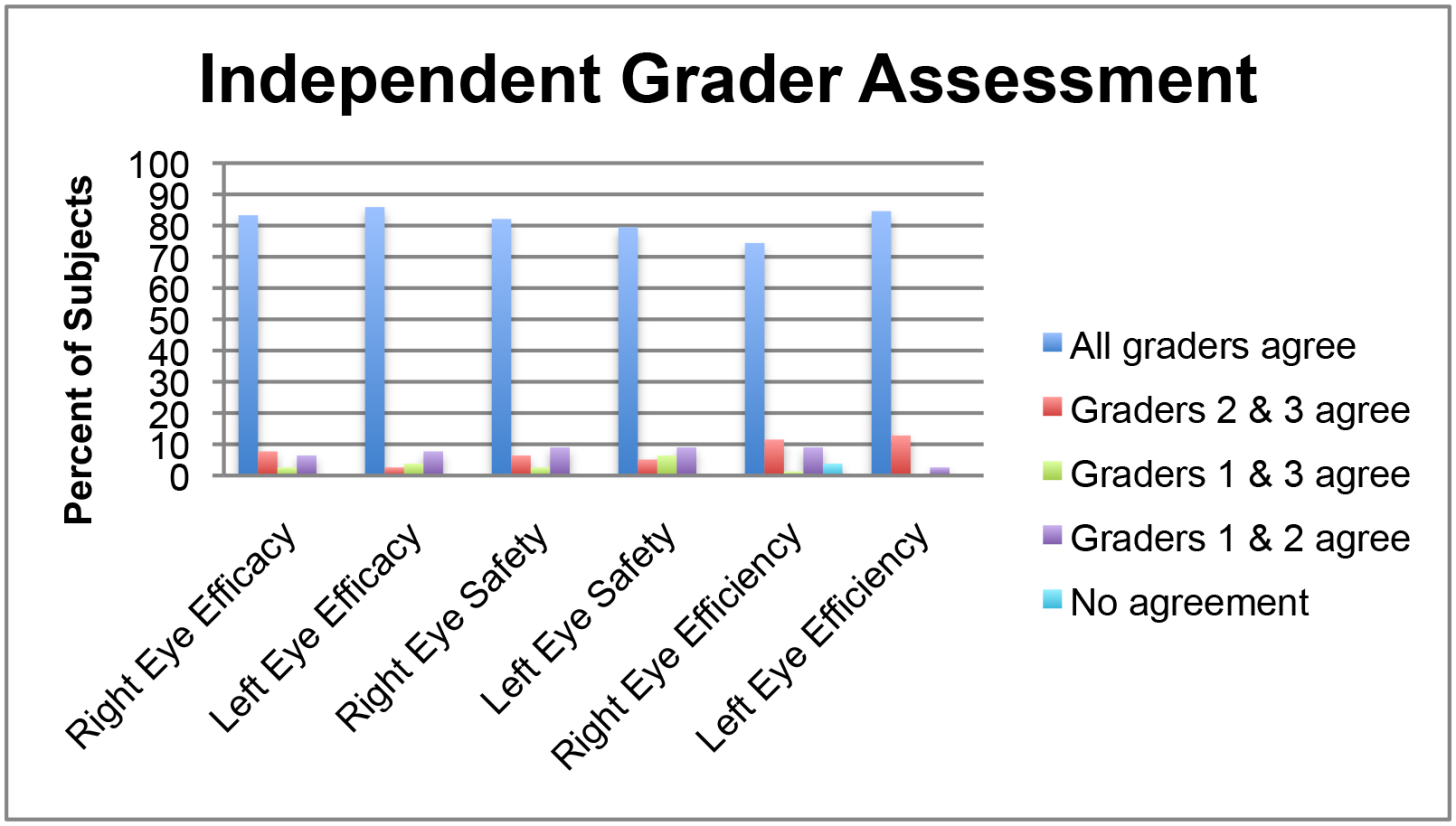
Mean Kappa Levels of Agreement among Three Masked Graders. Graphical representation of the agreement among graders for efficacy, safety and efficiency, separated by right and left eyes.

All raw data are included in a supporting information Excel spreadsheet (S1 Raw Data). Kappa statistics describing the level of agreement among the graders are summarized in Table 1. Fig 1 graphically demonstrates the proportion of eyes for which there was agreement among graders.

**Table 1:** Mean Kappa Levels of Agreement among Three Masked graders.

	n = 78	n = 78	n = 78
	Average Kappa—(95% CI)	Average Kappa—(95% CI)	Average Kappa (95% CI)
	Right Eye	Left Eye	Both Eyes Combined
Efficacy (Simple Kappa)	0.60 (0.36–0.84)	0.68 (0.47–0.89)	0.64 (0.42–0.87)
Safety (Simple Kappa)	0.75 (0.60–0.89)	0.71 (0.56–0.87)	0.73 (0.58–0.88)
Efficiency (Weighted Kappa)	0.55 (0.37–0.73)	0.68 (0.47–0.88)	0.62 (0.42–0.81)

Efficacy: The mean kappa level of agreement for efficacy was 0.60 (95% CI, 0.36–0.84) for the right eye and 0.68 (95% CI, 0.47–0.89) for the left eye. When combining the average of the right and left eyes, the mean kappa level of agreement for efficacy was 0.64 (95% CI, 0.42–0.87). The range of grader scores of percentages of patients with successful eye drop instillation onto the ocular surface was 79.5–87.2% and 74.4–87.2% for the right and left eye, respectively.

*Safety*: The mean kappa level of agreement for safety was 0.75 (65% CI, 0.60–0.89) for the right eye and 0.71 (95% CI, 0.56–0.87) for the left eye. When combining the average of the right and left eyes, the mean kappa level of agreement for safety was 0.73 (95% CI, 0.58–0.88). The range of grader scores of percentages of patients making no contact between the bottle tip and ocular or skin surface was 46.2–66.7% and 57.3–74.4% for the right and left eye, respectively.

*Efficiency*: The mean kappa level of agreement for number of drops instilled was 0.55 (95% CI, 0.37–0.73) for the right eye and 0.68 (95% CI, 0.47–0.88) for the left eye. When combining the average of the right and left eye, the mean kappa level of agreement for efficiency was 0.62 (95% CI, 0.42–0.81). The range of grader scores of percentages of patients using 1 drop per instillation was 64.1–76.9% and 64.1–89.7% for the right and left eye, respectively.

## Discussion

We propose a standardized system for evaluating eye drop self-instillation technique using three domains. *Efficacy* is a determination of whether an eye drop made contact with the ocular surface. *Safety* is a determination of whether the tip of the bottle made physical contact with the ocular surface or eyelid or facial skin. *Efficiency* is a determination of the number of eye drops expressed from the medication bottle. Using this evaluation system, we evaluated the eye drop instillation technique of both eyes of 78 patients. The kappa levels of agreement combined for both eyes for efficacy (0.64), safety (0.73), and efficiency (0.62) demonstrated substantial agreement among graders, suggesting the assessment of video recordings to evaluate eye drop self-instillation technique is valid and reasonably reproducible.

Prior studies have used video recording to assess whether patients successfully self-instill eye drops [9, 11–13]. Stone, et al [9] used a single grader observing video recordings of glaucoma patients and found that 14 of 64 (21.9%) and 36 of 117 (30.8%) patients successfully instilled a single drop into the eye without touching a 15 ml and 2.5 ml bottle tip to the eye, respectively. Hennessy, et al [11] also used a single grader and found that 39% of patients successfully instilled a single drop into the eye without touching the bottle tip to the eye. Since only one grader assessed the video recordings in these studies, the reproducibility of the assessments of the recordings is unknown.

**Fig 2:**
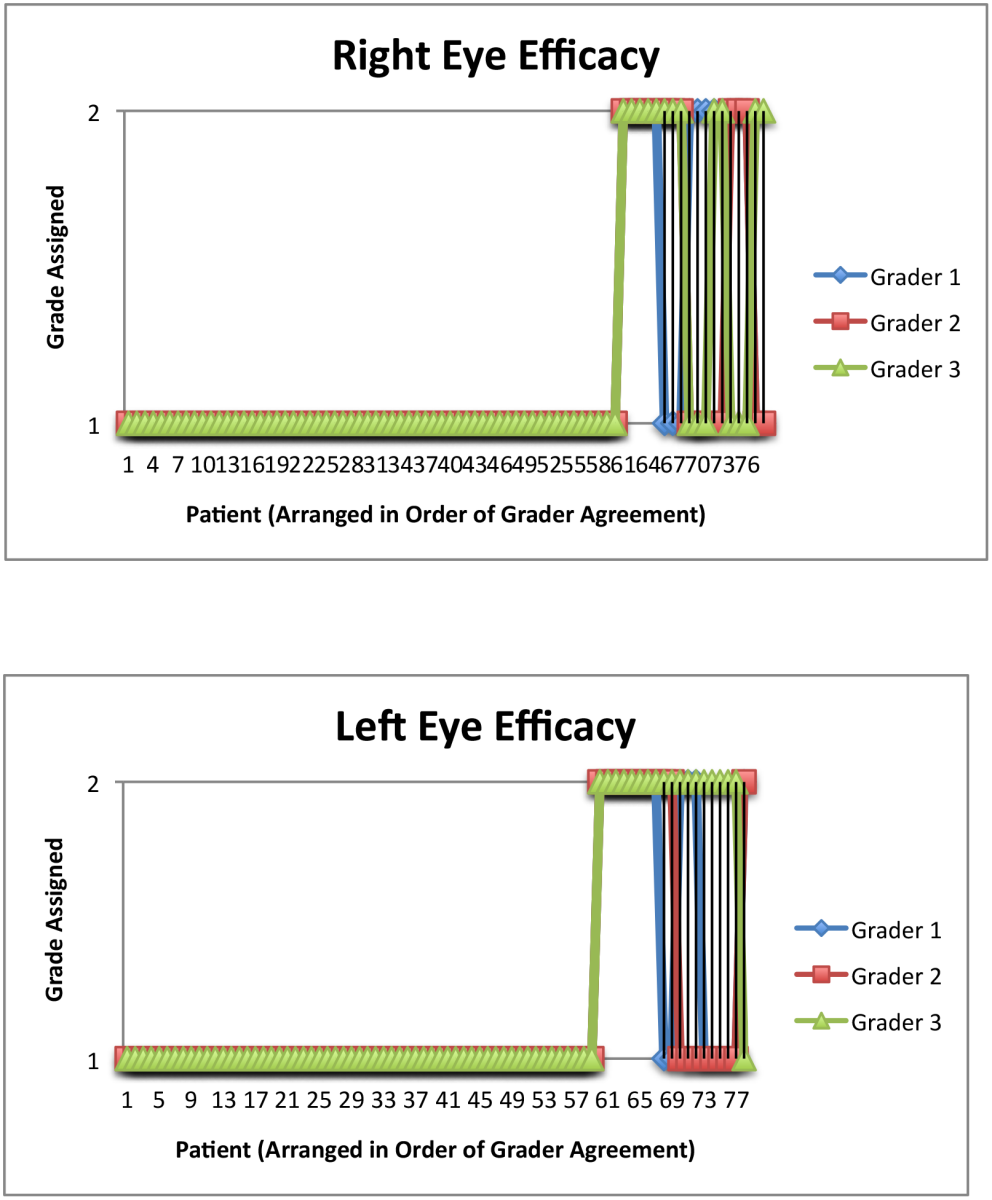
a: Efficacy Grades for the Right Eye. b: Efficacy Grades for the Left Eye. Fig 2a and 2b show the efficacy ratings that were assigned to each patient for the right and left eyes, respectively, by each grader. All three graders agreed for 83.3% of right eyes and 82.1% of left eyes. A score of 1 indicates successful instillation of the eye drop. A score of 2 indicates an eye drop did not land on the ocular surface.

The results of the current study are similar to those reported previously with respect to successful self-instillation of eye drops. Stone, et al [9] found that 96 of 116 (82.8%) with a 15 mL bottle and 48 of 64 (75.0%) with a 2.5 mL bottle successfully instilled an eye drop onto the ocular surface, compared to 60 of 78 (76.9%) and 59 of 78 (75.6%) for the right and left eye, respectively in the current study (Fig 2a and 2b). Hennessy, et al [12] similarly reported that 71% of glaucoma patients successfully instilled a drop onto the ocular surface.

**Fig 3:**
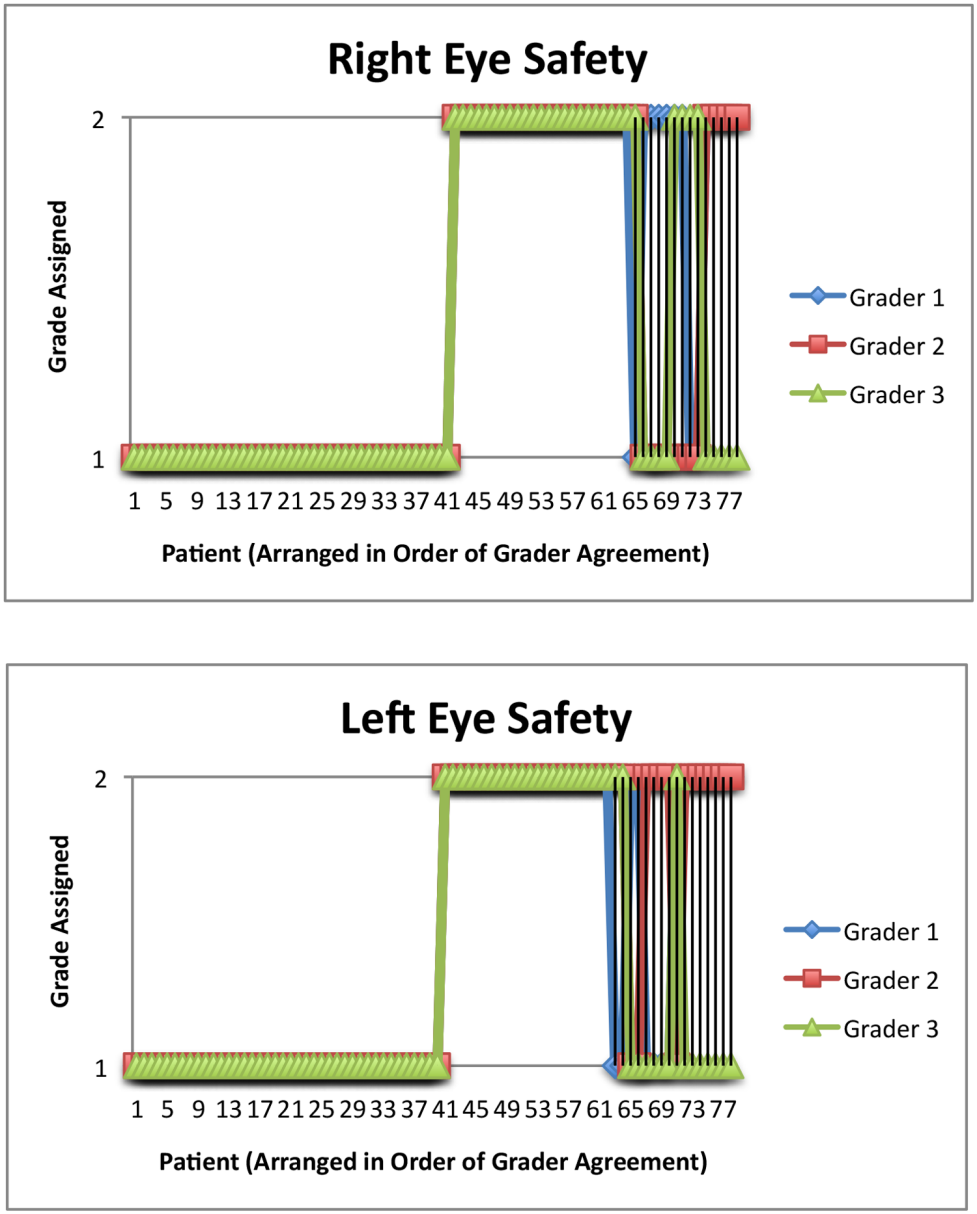
a: Safety Grades for the Right Eye. b: Safety Grades for the Left Eye. Fig 3a and 3b show the safety ratings that were assigned to each patient for the right and left eyes, respectively, by each grader. All three graders agreed in 82.1% of right eyes and 79.5% of left eyes. A score of 1 indicates the bottle tip did not touch the ocular surface or eyelid. A score of 2 indicates the bottle tip touched the ocular surface, eyelids, eyelashes, or cheek.

Of the three parameters, patients were least successful with safety: 23 of 78 (29.5%) and 22 of 78 (28.2%) patients touched the bottle tip to the ocular adnexae of the right and left eyes, respectively, in this study (Fig 3a and 3b). This compares to 34% of patients touching the bottle tip to the eye or eyelashes and 52% of patients touching the face with the bottle tip in a previous report by Sleath, et al [13]. In Hennessy, et al, 33% of glaucoma patients video recorded self-administering eye drops [12] made contact between the eye and bottle tip. This highlights the importance in educating glaucoma patients of the need to avoid touching the bottle tip to the ocular surface, thus reducing the risk of trauma or infection.

**Fig 4:**
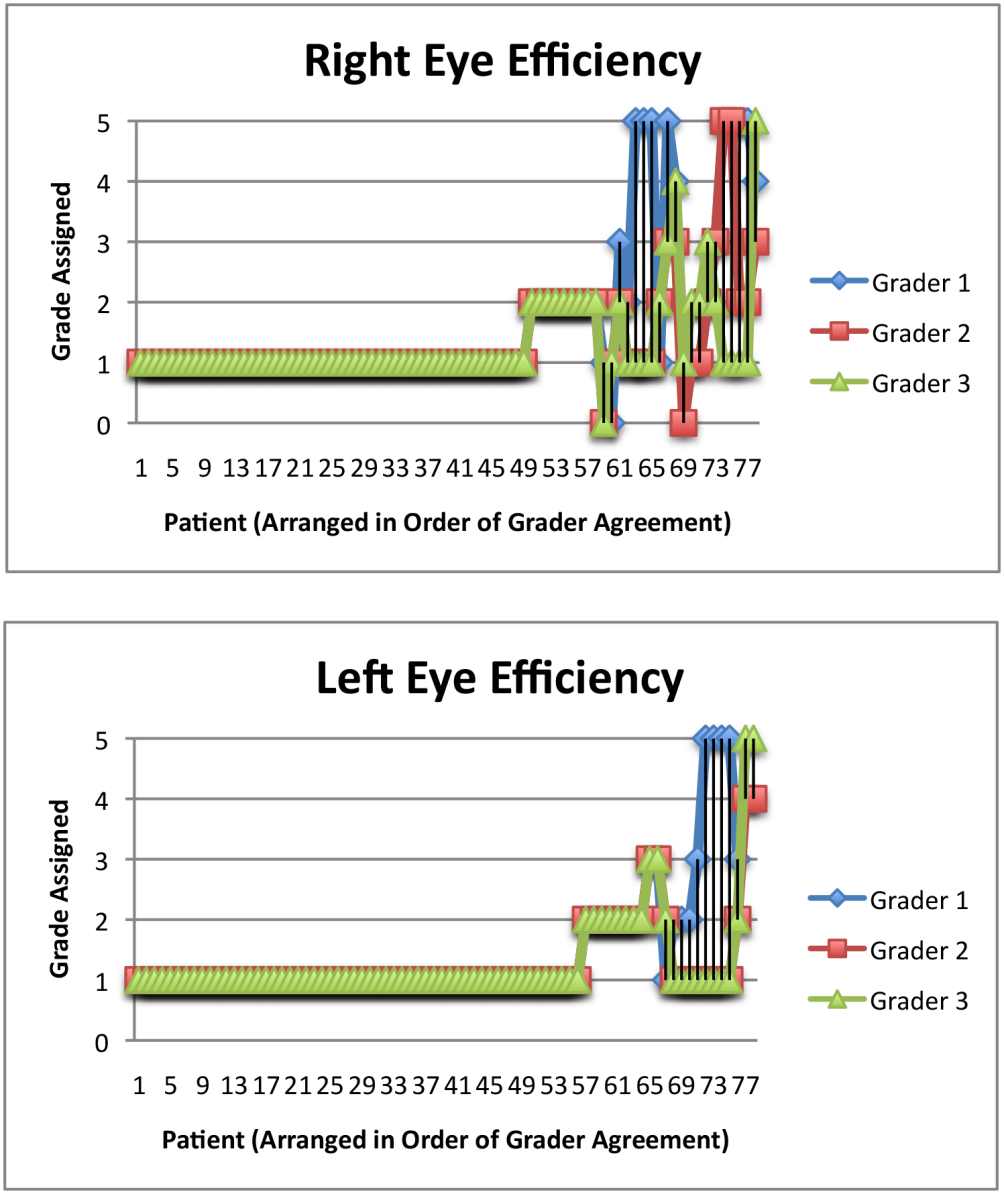
a: Efficiency Scores for the Right Eye. b: Efficiency Scores for the Left Eye. Fig 4a and 4b show the graders’ assessments of the number of eye drops expressed from the bottle for the right and left eyes, respectively. All three graders gave a score of 1 (only 1 eye drop was instilled) for 62.8% of right eyes and 71.8% of left eyes. Scores of 0–5 were allowed, with 5 indicating a continuous stream was expressed.

Hennessy, et al reported 147 of 204 (72.1%) used one drop [11], compared to 49 of 78 (62.8%) and 56 of 78 (71.8%) in the right eye and left eye, respectively for the current study (Fig 4a and 4b; Table 2). 25 of 64 (39.1%) and 55 of 116 (47.4%) of patients in the Stone, et al [9] study used only eye drop per instillation using the 15 ml and 2.5 ml bottle respectively. They also reported 19 of 64 (29.7%) and 26 of 116 (22.4%) of patients instilling a continuous stream of fluid, which is much higher compared to our study.

**Table 2:** Grades given for Efficacy.

Efficacy	Number of Eye Drops Instilled (# of patients (%))					
	1	2	3	4	5	Disagreement Among Graders
**Right Eye**	**49 (62.8)**	**9 (11.5)**	**0 (0)**	**0 (0)**	**0 (0)**	**20 (25.6)**
**Left Eye**	**56 (71.8)**	**8 (10.3)**	**2 (2.6)**	**0 (0)**	**0 (0)**	**12 (15.4)**

All three graders gave a score of only 1 drop instilled for 62.8% and 71.8% of the patients for the right and left eye, respectively.

A limitation of this study is the fact the video recordings were made using standard lighting conditions and amateur videographers. Therefore, the video recordings may not have been of the highest possible quality, although this may be more representative of the methodology and typical conditions utilized by most investigators. The artificial tear bottle used in the study may differ significantly from the glaucoma medication bottle used by the participants at home. Some video recordings may have been blurry or may not have clearly captured the entire process of eye drop instillation. Graders were required to make forced choice decisions, so it is possible that agreement could have been higher if recordings of poor quality were excluded from the analysis. Although we did not capture information on the quality of the recordings, we believe the vast majority were of high quality. Kappa statistics appropriately adjust for statistical chance agreement and therefore, are lower than the actual agreement observed in the data. In this study, the average kappa statistics were 30% lower than raw agreement for efficacy and efficiency, and 16% lower for safety. Therefore, kappa statistics, although statistically appropriate, underestimate the actual agreement.

This is the first study that analyzes the validity of using a three-component system for grading proper self-instillation of eye drops: efficacy, safety, and efficiency. As one of the main treatments for glaucoma is topical medications to control intraocular pressure, glaucoma patients should ideally know how to properly instill a single eye drop onto the ocular surface. This would not only allow for better control of intraocular pressure, but would minimize excessive costs of using more than one drop per instillation [2–3, 5–6, 8]. Furthermore, patients must not contaminate the bottle tip to avoid introducing pathogens that could lead to infections [7]. Video recording is advantageous in that it guides both researchers and clinicians in determining whether patients are properly administering eye drops. Using the three parameters of efficacy, safety, and efficiency may facilitate better understanding of the barriers to proper eye drop instillation. Use of this validated, standardized method to grade eye drop self-instillation technique will allow investigators to compare results from different studies in a more meaningful way which will help advance research in this field.

## Supporting Information

S1 Raw Data Excel spreadsheet with raw data for 78 participants. (XLSX)
